# EXPANDING THE ENGAGEMENT OF OLDER PERSONS IN CLIMATE CHANGE ACTION

**DOI:** 10.1093/geroni/igad104.0691

**Published:** 2023-12-21

**Authors:** Karl Pillemer, Matthew Luebke

**Affiliations:** Cornell University, Ithaca, New York, United States; Cornell University, Ithaca, New York, United States

## Abstract

Environmental volunteerism and civic engagement have been found to promote physical and mental well-being among older adults as well as helping communities address environmental issues. Despite the growing need for citizen action to address disasters and other climate change effects, however, little is known about how to promote such engagement. At present, few climate change organizations actively recruit or provide specific opportunities for older persons. Academics and policymakers have focused on how older people are particularly susceptible to the impact of climate change. Limited attention has been paid, however, to the ways in which older people can be actively involved on their own behalf in climate change mitigation, including engagement in disaster preparation and response. In this presentation, I review barriers to widespread mobilization of older adults in climate change action, including their lower levels of environmental concern and their systematic lack of access to opportunities for climate change activism. I report on findings from a recent international survey of local and regional organizational efforts to engage older persons in climate change prevention and mitigation. This survey identified successful models for mobilizing older persons to address climate change mitigation, and in particular in low- and middle-resource countries. Recommendations for effective program design to bring older persons more fully into climate change action are presented, as well as ways environmental organizations can expand their focus to include systematically older persons in their activities.

